# Proteomic insights into mental health status: plasma markers in young adults

**DOI:** 10.1038/s41398-024-02751-z

**Published:** 2024-01-24

**Authors:** Alexey M. Afonin, Aino-Kaisa Piironen, Izaque de Sousa Maciel, Mariia Ivanova, Arto Alatalo, Alyce M. Whipp, Lea Pulkkinen, Richard J. Rose, Irene van Kamp, Jaakko Kaprio, Katja M. Kanninen

**Affiliations:** 1 A.I. Virtanen Institute for Molecular Sciences, University of Eastern Finland, Kuopio, Finland; 2 Institute for Molecular Medicine Finland (FIMM), HiLIFE, University of Helsinki, Helsinki, Finland; 3 Department of Psychology, University of Jyvaskyla, Jyvaskyla, Finland; 4 Department of Psychological & Brain Sciences, Indiana University, Bloomington, IN USA; 5 Centre for Sustainability, Environment and Health, National Institute for Public Health and the Environment, Bilthoven, the Netherlands; 6 Department of Public Health, University of Helsinki, Helsinki, Finland

**Keywords:** Psychology, Biomarkers

## Abstract

Global emphasis on enhancing prevention and treatment strategies necessitates an increased understanding of the biological mechanisms of psychopathology. Plasma proteomics is a powerful tool that has been applied in the context of specific mental disorders for biomarker identification. The p-factor, also known as the “general psychopathology factor”, is a concept in psychopathology suggesting that there is a common underlying factor that contributes to the development of various forms of mental disorders. It has been proposed that the p-factor can be used to understand the overall mental health status of an individual. Here, we aimed to discover plasma proteins associated with the p-factor in 775 young adults in the FinnTwin12 cohort. Using liquid chromatography–tandem mass spectrometry, 13 proteins with a significant connection with the p-factor were identified, 8 of which were linked to epidermal growth factor receptor (EGFR) signaling. This exploratory study provides new insight into biological alterations associated with mental health status in young adults.

## Introduction

Mental health issues are increasingly becoming a major concern globally [1, 2]. In fact, the World Health Organization estimates that about one in every eight people across the globe suffer from a mental health disorder, making these disorders the primary cause of a reduced quality of life [1]. The recent COVID-19 pandemic has notably exacerbated mental health issues, particularly among young adults aged 18–29. During the pandemic, the number of young adults experiencing depression symptoms more than doubled in numerous European countries [3].

Despite the significant impact of mental health diseases on daily life and their considerable economic cost, these conditions often go undiagnosed and untreated [1, 3]. This highlights the pressing need for early detection of individuals at high risk for psychopathology, targeted preventative measures, and improvements in diagnostic procedures and treatments.

Although different mental disorders may have unique symptoms, they have been shown to share commonalities in terms of underlying biological, psychological, and social factors [4]. The p-factor, also known as the “general psychopathology factor,” is a concept in psychopathology suggesting that there is a common underlying factor that contributes to the development of various forms of mental disorders [5, 6]. It has been proposed that this single latent factor can encapsulate individuals’ proclivity to develop all forms of psychopathology included within the broad internalizing, externalizing, and thought disorder dimensions [7]. The p-factor is analogous to the general factor in intelligence (called the g-factor), which summarizes the observation that individuals who do well on one type of cognitive test tend to do well on all other types of cognitive tests [5, 8]. Other factors, such as a general factor of personality (GFP) and a general factor of personality disorder (g-PD), have been previously shown to have a high correlation with the p-factor [9]. At the individual level, the p-factor reflects meaningful differences between persons on a single dimension that represents the tendency to experience psychiatric problems as persistent and comorbid; that is, high p-factor individuals experience difficulties in regulation/control when dealing with others, the environment, and the self [4, 5, 10].

Previous studies have shown the p-factor to be connected to brain functioning in adolescents, with higher p-factor scores associated with diminished activation of multiple brain zones during executive tasks [11]. Importantly, some studies have reported that the p-factor may be a stronger predictor of mental health outcomes than specific diagnoses of mental disorders [12]. A recent study showed that the p-factor was associated with poorer performance on the simple reaction time task and the inspection time task, with speed of processing being a common correlate of psychopathology factors [13]. Likewise, Pulkkinen [14] has shown that low emotion and behavior regulation observed as externalizing and internalizing problems in children are negatively associated with the executive functions of the forebrain for inhibition and updating (containing working memory and shifting). This suggests that the p-factor could be used to better understand the overall mental health status of an individual, rather than just focusing on individual diagnoses.

Biomarker discovery has gained traction in recent years as researchers seek to uncover the biological underpinnings of mental health conditions [15, 16]. The development of “omics” technologies and state-of-the-art analytical methods have increased interest in the capabilities of plasma proteomics in biomarker discovery. LC-MS/MS-based proteomics provides a global snapshot of protein expression patterns that reflect physiological and pathological states [17], making comprehensive analysis of the plasma proteome possible [18]. This has enabled the simultaneous detection and quantification of thousands of proteins, expediting biomarker discovery efforts and reducing the time and resources required for this process. This holistic view of proteomics allows for the unbiased discovery of novel biomarkers, with less need for prior knowledge of target proteins. This feature is particularly important in cases where the biology of the process is not yet fully understood or when new, unforeseen biomarkers are needed for improved diagnostic or prognostic applications [19].

Proteomics approaches have been utilized for the identification of protein signatures associated with specific psychological disorders [20–22]. For example, several growth factors (BDNF, VEGF, NGF) and cytokines (IL-1β, IL-6, IFN-α) have been linked to depression [23]. Moreover, a recent multi-omics study reported reduced apolipoprotein levels and an increase in complement effector proteins in the plasma of schizophrenia (SCZ) patients [24]. However, proteomics analyses have not previously been combined to studies of the p-factor for identification of markers associated with overall mental health status.

The FinnTwin12 (FT12) cohort, a longitudinal study of Finnish twins born between 1983 and 1987, has a multitude of data and biological samples [25, 26]. As a valuable resource for exploring biological processes involved in mental health problems, we explored the connection between the p-factor, previously reported in [27], and plasma proteomics among young adults from this cohort.

## Methods

### Cohort description

The FT12 cohort is a longitudinal population-based cohort of Finnish twins born 1983–1987 collected to investigate behavioral development and health habits [25, 26]. Initially, twins and their families were identified using the Finnish Central Population Registry, and questionnaire collection occurred for all participants in the cohort at ages 11/12, 14, 17, and 22. The baseline response rate was 87% (*N* = 5600 twins) and has remained high (response rate range: 85–90%). At age 14, a subset of the twins (from 1035 families) was more intensively studied, including psychiatric interviews and additional questionnaires (ages 14 and 22), as well as blood plasma samples (age 22). The “age 22” assessment wave of these more intensively studied twins involved 1347 individuals (mean age = 22.4 years, SD = 0.70; response rate 73.0%), 779 of whom attended in-person assessments and provided venous blood plasma samples. The blood samples were collected after overnight fasting, which involved abstaining from alcohol and tobacco since the night before sampling. Plasma was immediately extracted and stored at −80 °C [27].

### p-factor calculation

In FT12, behavioral and emotional characteristics were measured at all data collection waves. The modified Multidimensional Peer Nomination Inventory (MPNI) measure aimed at observing individual differences in emotion and behavior regulation was used. It is an extension of the measure [28] used in the Jyväskylä Longitudinal Study of Personality and Social Development in which the development of the same individuals has been followed from age 8–50, with findings that low self-regulation is associated with social and psychological dysfunction [14]. The MPNI scale has been previously factor analyzed with three main factors termed Behavior Problems, Emotional Problems, and Adjustment [28].

The MPNI was collected in FT12 at ages 12, 14, and 17, from different raters (7 in total): parents (age 12), teachers (age 12 and 14), twin children themselves (age 14 and 17), and the child’s co-twin (age 14 and 17). The measure includes subscales of the externalizing problem dimension: aggression (6 items [for MPNI ages 12, 14, 17]), hyperactivity-impulsivity (7 items [MPNI ages 12, 14], 6 items [MPNI ages 17]), and inattention (4 items [MPNI ages 12, 14, 17]), as well as for the internalizing problem dimension; depression (5 items [MPNI ages 12, 14], 2 items [MPNI ages 17]), social anxiety (2 items [MPNI ages 12, 14], 3 items [MPNI ages 17]), and 1 item for victimization (MPNI ages 12, 14, 17). Each MPNI item (e.g., “Is restless, unable to sit still”) has four response choices (from “not observed in the child” to “clearly observable in the child”, scored 0–3 respectively). The MPNI p-factor score was created by combining all the items of the “externalizing” and “internalizing” dimensions together into a sum score, with at most 2 missing items allowed. Missing items were imputed based on the mean of the remaining items, with less than 3% of twins having missing items. A composite “combined” p-factor score was created using the p-factor scores of all seven of the abovementioned available MPNI ratings (Cronbach’s alpha=0.76), because we know that ratings from different raters are not highly correlated, however, they can impart unique information [29–31]. Each of the seven scores were standardized as *z* scores, and then we took the mean of available scores. The p-factor for the FT12 cohort was previously created and analyzed in relation to metabolites in [27]. Eleven twins had no overall p-factor score, leaving 775 twins. Of them, 505 (65%) had been scored by all raters at all times, while 194 (25%) had only one rater value missing, the remaining 10% having scores from 2–4 raters. To examine the dimensionality of combining the seven individual p-factor scores, we performed a factor analysis on the subset of participants who had been rated on all measures. The factor analysis indicated one major factor, with the first eigenvalue associated with the first factor having a value well over one. A correlation analysis was performed for the newly calculated score with the sum scale based on all seven p-factors, showing a high correlation coefficient of 0.983. The composite “combined” p-factor score was used for the subsequent analyses.

The predictive power of the p-factor was tested using the data on the psychiatric interviews of the twins at age 22. Using a logistic regression model for MDD, p-factor score, adjusted for sex, predicts MDD reasonably well with an area under the receiver operating characteristic curve (ROC AUC) of 0.67.

### High-abundance protein depletion

Albumin accounts for 50%, and the top 22 proteins account for 99% of plasma proteins by weight in human plasma samples [19]. Therefore, the depletion of high-abundant proteins is essential to the identification and analysis of low-abundant proteins. A commercial kit (High Select™ Top14 Abundant Protein Depletion Mini Spin Columns, cat. Number: A36370, ThermoScientific) was used to deplete the 14 most abundant proteins from plasma before the proteomic analyses. The depleted proteins were human serum albumin (HSA), albumin, IgG, IgA, IgM, IgD, IgE, kappa and lambda light chains, alpha-1-acidglycoprotein, alpha-1-antitrypsin, alpha-2-macroglobulin, apolipoprotein A1, fibrinogen, haptoglobin, and transferrin, according to manufacturer’s manual. Briefly, 10 µL of total plasma was added to the mini spin columns and incubated for 10 min while rotating, followed by centrifugation of the columns (1,000 × *g*) for 2 min. The filtrate was collected in 2 ml plastic tubes and stored at −20 °C until preparation for mass spectrometry proteomic analyses, which were performed at the Turku Proteomics Facility in Finland supported by Biocenter Finland.

### Protein precipitation and digestion

The proteins of 786 depleted plasma samples were acetone precipitated and subjected to in-solution digestion according to standard protocol at the Turku Proteomics Facility, Turku, Finland (https://bioscience.fi/). After digestion, peptides were desalted with a Sep-Pak C18 96-well plate (Waters), evaporated to dryness, and stored at −20 °C.

### Mass spectrometry analysis

Digested peptide samples were dissolved in 0.1% formic acid, and the peptide concentration was determined with a NanoDrop device. For data-independent acquisition (DIA) analysis, 500 ng of peptides were injected and analyzed in a random order, determined with the Excel rand() function. Wash runs were submitted between each sample to reduce potential carry-over of peptides. The Liquid Chromatography-Electrospray Ionization-Mass Spectrometry (LC-ESI-MS/MS) analysis was performed on a nanoflow HPLC system (Easy-nLC1000, Thermo Fisher Scientific) coupled to a Q Exactive HF mass spectrometer (Thermo Fisher Scientific, Bremen, Germany) equipped with a nano-electrospray ionization source. Peptides were first loaded on a trapping column and subsequently separated inline on a 15 cm C18 column (75 μm x 15 cm, ReproSilPur 3 μm 120 Å C18-AQ, Dr. Maisch HPLC GmbH, Ammerbuch-Entringen, Germany). The mobile phase consisted of water with 0.1% formic acid (solvent A) or acetonitrile/water (80:20 (v/v)) with 0.1% formic acid (solvent B). A 50 min from 5% to 35% solvent B gradient was used to elute peptides. Samples were analyzed by a DIA LC-MS/MS method. MS data was acquired automatically by using Thermo Xcalibur 4.1 software (Thermo Fisher Scientific). In the DIA method, a duty cycle contained one full scan (400–1000 m/z) and 25 DIA MS/MS scans covering the mass range 400–1000 with variable width isolation windows.

### Protein identification and quantification analysis

Data analysis consisted of protein identification and label-free quantifications of protein abundances. Data was analyzed using Spectronaut software (Biognosys; version 17.0.2). The direct DIA approach was used to identify proteins. The main data analysis parameters in Spectronaut were: (i) Enzyme: Trypsin/P; (ii) up to 2 missed cleavages; (iii) Fixed modifications: Carbamidomethyl (cysteine); (iv) Variable modifications: Acetyl (protein N-terminus) and oxidation (methionine); (v) Precursor FDR Cutoff: 0.01; (vi) Protein FDR Cutoff: 0.01; (vii) Quantification MS level: MS2; (viii) Quantification type: Area under the curve within integration boundaries for each targeted ion; (ix) Protein database: Homo sapiens Swiss-Prot reference proteome (Uniprot release 2022_01_07_HUMAN) [32], Universal Protein Contaminant database [33].

### Raw data drift and batch correction

Protein abundances were analyzed by LC-MS/MS in three separate experimental runs or batches. Since the number of samples in each batch was relatively large, the data was normalized before further analysis, and batch effects were removed. For the ease of comparing the LC-MS/MS runs, 10 of the samples were analyzed in 2 out of the 3 runs. For the raw data analysis, we extracted the data from the .sne file using the iq export scheme [34]. The data used for normalization was the raw peak area of the peptide groups. These values were used in the further analyses.

The raw data investigation pre-processing and statistical analyses were performed in the R (version 4.2.1.) environment (R Core Team, 2022). The signal drift and the observed batch effect were corrected using the proBatch (v. 1.13.0) [35] package. The median abundance plots showed the samples forming four distinct groups, identical to the batches of instrument runs (Supplementary Fig. 1A). The figure also shows pronounced signal drift in the third and fourth batches. These effects were corrected for using the proBatch pipeline. After the batch effect correction, no significant drift or batch effect could be seen (Supplementary Fig. 1B).

### Bioinformatic analysis

After drift and batch correction, the fastMaxLFQ method from the iq package (v. 1.9.10) [34] was used to transform the peptide abundancies into protein abundance values. The contaminant proteins and the proteins depleted in the sample pre-processing step were removed from the analysis. Only the identified proteins with quantified abundance levels in at least 80% of the samples were used in further analyses. Missing values remaining in the dataset were imputed using the Sample Minimum method [36]. As an additional sensitivity test, the same modeling was performed using the proteins present in 20, 40, 60, 80, and 98% of the samples, to ensure that the modeling was robust, and that the exclusion of rare proteins did not skew the analysis.

The connection between the p-factor and the protein abundances was analysed using the limma [37] package (v. 3.54.2). Sex and age were included into linear models as covariates to ensure reported associations were not due to sex or age effects. Limma modeling was used to investigate the association of protein abundance with the p-factor using linear and non-linear modeling. The possible non-linear relationship between the p-factor and the protein abundance was investigated by using splines in limma [38]. A basis matrix for representing the family of piecewise-cubic splines with 5 nodes were generated using the *ns* function from the p-factor variable (Splines package v3.6.2), and was used in limma modeling, also including sex and age as covariates.

Moderate *F* test on the p-factor was carried out to assess the significance of non-linear associations of the protein abundance with the p-factor using the function lmFit and eBayes from the R limma package. *P* values for linear and non-linear modeling were corrected for multiple testing and the false discovery rate (FDR) was computed by using the Benjamini & Hochberg method [39], which were reported as q-values. The significance level considered in all analyses was 0.05. The linear effect size is reported as the log2-fold-change in expression that results from a unit (one standard deviation) change in p-factor.

The protein–protein interaction information for the significantly differentially abundant proteins was analysed using STRING database (v. 12.0) [40]. The enrichment analysis with Gene Ontology (Process, Function, and Component), KEGG and Reactome pathways, PubMed publications, UniProt Keywords, and PFAM/INTERPRO/SMART domain databases was performed using the STRINGdb package [41]. Result visualizations were performed using R and ggplot2 (v3.4.0) [42].

## Results

### Cohort characteristics

**Fig. 1:**
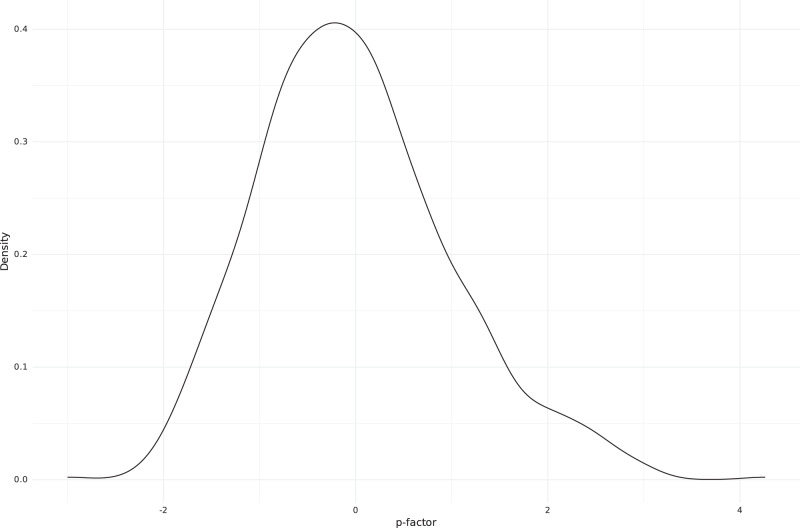
Distribution of p-factor values in the FT12 cohort. The density distribution of the standardized p-factor values of the participants.

The p-factor was calculated based on assessments by multiple raters at three different ages as described in the Materials and Methods. A combined p-factor value was available for 775 individuals (318 males and 457 females). The z-score-based p-factor distribution is presented in Fig. 1.

### Protein identification

MS-based proteomics successfully identified 1494 proteins (DIA spectrometry intensity values) in the FT12 cohort (*N* = 775) the list of found proteins is presented in Supplementary File 1. After the removal of values of proteins depleted in the sample pre-processing step, 1415 proteins were left, with a mean number of identified proteins of 835 per sample (SD = 48). Proteins present in at least 80% of the samples were used, leaving 571 proteins.

### Association of proteins with p-factor

The linear modeling showed 5 proteins inversely associated with the p-factor (Table 1). As the relationship between the altered proteins and p-factor is not known, the analysis was also performed using splines, which also made it possible to investigate non-linear relationships between the protein abundance and the p-factor. These analyses showed 14 proteins associated with the p-factor (Table 1). The relationships between the p-factor and the protein abundance for the significantly associated proteins are presented in Supplementary Figure 2.

**Table 1:** The plasma proteins significantly associated with the p-factor.

Protein ID	Gene name	Protein name	Linear effect size	Linear *p* value	Linear *q* value	Non-linear *p* value	Non-linear *q* value
Proteins with linear relationship with p-factor							
Q15828*	CST6	Cystatin E/M	−0.086	1.76E-04	0.024	8.43E-04	0.047
P98160	HSPG2	Heparan sulfate proteoglycan 2	−0.061	3.86E-05	0.006	1.27E-03	0.066
P23142	FBLN1	Fibulin-1	−0.065	2.49E-05	0.006	1.74E-03	0.075
P07911*	UMOD	Uromodulin	−0.145	1.19E-05	0.006	8.89E-05	0.013
Q9Y6R7*	FCGBP	Fc gamma binding protein	−0.082	8.11E-06	0.005	3.05E-05	0.007
Proteins with non-linear relationship with p-factor							
P04179	SOD2	Superoxide dismutase 2	–	3.90E-01	0.701	1.86E-06	0.001
Q9BY67	CADM1	Cell adhesion molecule 1	–	3.87E-02	0.329	4.24E-06	0.002
Q14126	DSG2	Desmoglein 2	–	8.53E-04	0.072	8.80E-05	0.010
Q8NBJ4	GOLM1	Golgi membrane protein 1	–	7.93E-01	0.866	2.68E-04	0.010
P07858	CTSB	Cathepsin B	–	1.22E-01	0.496	6.74E-05	0.010
Q86UN3	RTN4RL2	Reticulon-4 receptor-like 2	–	3.66E-01	0.675	8.59E-05	0.012
O75636	FCN3	Ficolin 3	–	9.16E-03	0.183	3.42E-04	0.026
P07942	LAMB1	Laminin subunit beta-1	–	9.11E-03	0.183	5.13E-04	0.035

^*^indicate proteins with both linear and non-linear relationship.

The sensitivity testing showed that 13 of 14 proteins were consistent across models, the S100 calcium binding protein A4 was significantly associated with the p-factor only when proteins missing in over 20% of samples were excluded. The results of additional analyses are presented in Supplementary Table 1.

### Functional enrichment and annotation

**Fig. 2:**
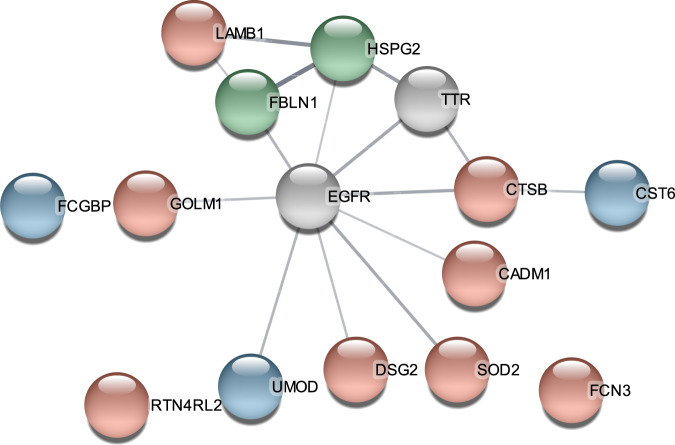
The result of STRING analysis of the proteins significantly associated with the p-factor. Line thickness indicates the strength of data support. Green circles denote proteins with a linear relationship to the p-factor, red circles with non-linear relationships, and blue circles representing proteins with reported linear and non-linear relationships.

The STRING protein–protein interaction networks functional enrichment analysis showed two connected clusters of proteins with: the first being cystatin-M (CST6) and cathepsin B (CTSB), the second containing laminin subunit beta-1 (LAMB1), basement membrane-specific heparan sulfate proteoglycan core protein (HSPG2), and fibulin-1 (FBLN1) (Fig. 2). Investigation of the first layer of the string network showed that nine of the significant proteins were linked specifically through the epidermal growth factor receptor (EGFR) and transthyretin (TTR) (Fig. 2). Both proteins were among the 636 proteins we investigated, though the q-values were above the significance threshold (*q* values for both EGFR and TTR were 0.066).

Enrichment analysis of function categories showed only the extracellular matrix structural constituent to be significantly enriched. Compartments, component, function, and tissue analyses showed significantly enriched terms, mostly connected to extracellular space and matrix, and cell–cell adhesion (Supplementary Table 2).

A connection to a disease of the CNS or other neurodegenerative diseases according to the Disease Ontology database was found for 6 of the 13 significant proteins [43], shown in Table 2.

**Table 2:** The disease ontology classification (STRINGdb).

Gene name	Disease ontology
LAMB1, FCN3, CTSB, HSPG2	Nervous system disease
FCN3, CTSB, HSPG2, SOD2	Neurodegenerative disease
FCN3, CTSB, HSPG2	Central nervous system disease

## Discussion

The field of plasma proteomics is rapidly gaining traction in the realm of biomedical research, particularly in studies relating to mental health. There is increasing evidence that alterations in plasma protein profiles are associated with major psychiatric conditions, including major depressive disorder (MDD), schizophrenia, psychotic disorders (PSD), and bipolar disorders (BD) [44–46]. This study presents the first report of differences in plasma protein levels associated with the p-factor in a population sample of young adults.

We found 13 plasma proteins associated with p-factor scores in young adults. All but the FCGBP protein were present in the Human Plasma Proteome Database [47, 48], FCGBP, however, was previously reported in serum samples [49]. Of these proteins, ten belonged to a protein network connected to EGFR, eight being directly connected to EGFR. The EGF-related signaling pathways have been previously linked to neurodevelopment [50], synaptic plasticity [51, 52], chronic pain [53], fear [54], as well as mental health diseases [44, 52, 55–58]. For example, altered EGFR signaling has been reported in MDD and BD patients in blood proteomics studies [44].

In addition to EGFR signaling, we observed the p-factor to be negatively associated with heparan sulfate proteoglycan 2 (HSPG2). Heparan sulfate proteoglycans (HSPG) are membrane proteins and a major component of extracellular matrices involved in many cellular processes, as they function as co-receptors for growth factors [59]. HSPG2, combined with CEP350 and SMAD5, was recently presented as a potential diagnostic biomarker for MDD [60]. Furthermore, the *HSPG2* gene was previously connected to antipsychotic-induced adverse effects such as tardive dyskinesia [61, 62], specifically in SCZ patients [63], and the maintenance and repair of the blood-brain barrier in mice [64]. Moreover, downregulation of *HSPG2* and a depressive-like phenotype were revealed in mouse models of chronic mild stress and impaired glutamate function [65].

We also report a negative association with fibulin-1 (FBLN1) and the p-factor. The FBLN1 gene is connected to central nervous system development [66, 67] and modulation of neurotrophic activities of amyloid precursor protein in cultured rodent neural stem cells [68]. So far, little is known about the possible connection of FBLN1 to mental health. Shin [69]. reported decreased FBLN1 plasma protein levels in MDD patients compared to BD patients and healthy controls. The model proposed in that study also contained Fc gamma binding protein (FCGBP), which was reported to be significantly higher in BD patients compared to MDD patients but not in healthy controls. In our study, FCGBP was negatively associated with the p-factor. Additionally, increased plasma protein abundance of desmoglein 3 (DSG3) was reported in MDD patients and reduced abundance in BD patients compared to healthy controls [69]. DSG3 is a protein belonging to the same desmosomal cadherin family as the DSG2 reported in this study, which had a non-linear association with the p-factor with increased abundancies in the middle part of the p-factor scale. DSG2 was previously shown to have a similar function as DSG3 and was also shown to compensate for DSG3 in DSG3^−^ mouse models [70]. The Shin et al. paper investigated BD, which is classified as a Thought Disorder factor, and MDD, which is classified as an Internalizing factor [5], so the differences in the abundance changes observed compared to our work, where we used the combined p-factor, are to be expected. Laminin subunit beta-1 (LAMB1) was associated with the p-factor in this study. A polymorphism in *LAMB1* gene has been earlier associated with autism severity [71], neural development of embryonic stem cells [72] and pain sensitivity in mice [73]. *LAMB1* is expressed during the early development of nervous system [71] and in the hippocampus in the mature brain [74]. In rats, LAMB1 showed negative regulation of spatial learning through the inhibition of the ERK/MAPK-SGK1 signaling pathway in the hippocampus [74]. Furthermore, loss of LAMB1 in the anterior cingulate cortex was found to increase pain sensitivity and be associated with anxiety- and depressive-like behavior in mice [73].

Cathepsin B (CTSB) was identified here with a non-linear relationship to the p-factor. Moon *et al*. suggested CTSB as a mediator of exercise-induced effects on brain health by enhancing the expression of neurotrophins [75]. Exercise was found to increase plasma CTSB levels in monkeys and humans [75], but a 20-week exercise intervention in children did not find any significant connection between CTSB and brain health outcomes [76]. Additionally, CTSB has been connected to brain-related functions in several mice studies [77–79]. For example, a mouse model for chronic social stress revealed increased activity of cathepsin В in the hypothalamus and nucleus caudatus with depressive-like behavior [79]. Contrarily, decreased cathepsin B activity was found after acute emotional stress in mice [77]. CTSB shows a potential mediator role in the brain induced by physical and mental stressors, which should be further investigated.

Other proteins significantly associated with the p-factor and directly connected to EGFR in our study were golgi membrane protein 1 (GOLM1), superoxide dismutase 2 (SOD2), and uromoduline UMOD. Increased *GOLM1* gene expression was found in soldiers with PTSD [80]. This effect occurs through the activation of ErbB4-BDNF signaling pathway [81, 82]. Particularly strong evidence supports the role of the Neuregulin-1 (NRG1)-ErbB4 signaling on synaptic plasticity [51, 52]. Neuregulins are a family of epidermal growth factor-related proteins acting on the ErbB tyrosine kinase receptors [51, 52]. SOD2 was found to play a role in neurodegenerative disease according to the Disease Ontology database, a polymorphism in the *sod2* gene was associated with differences in white matter microstructure and suboptimal brain aging [83]. For the uromodulin (UMOD) proteins and CST6 gene, no previous connection to mental health problems was reported.

We observed associations of plasma reticulon-4 receptor-like 2 (RTN4L2), and ficolin 3 (FCN3) with the p-factor. Reticulon-4 receptors (RTN4R), also known as NogoRs, are surface proteins expressed in neurons [84]. RTN4Rs are involved in synaptogenesis and inhibition of axonal and dendrite growth, and, thus neuronal plasticity [84, 85]. Human genetics studies have revealed the linkage between Nogo receptors and SCZ [85–87]. For example, a rare variant in *RTN4R*, affecting the formation of growth cones in vitro, was associated with SCZ [87]. The role of RTN4Rs in SCZ seems to be mediated by neurodevelopmental and myelin-related abnormalities [85]. However, further studies are needed to clarify the exact role of RTN4Rs in mental health. Interestingly, ficolin activation was negatively associated with severity of SCZ [88], and in our recent study, the plasma abundance of ficolin 2, a similar protein, was found to be positively correlated with the Strength and Difficulties Questionnaire (SDQ) score in adolescents.

Half of the proteins found to be significant in this study were connected to the extracellular matrix. HSPG2, FBLN1, and LAMB1 were also strongly connected to each other, according to the STRING database, being structural components of the basal membrane, specifically in the brain. Coupled with proteins related to neuronal plasticity, proteins identified in this study may be potentially connected to the previously noted inverse relationship between the p-factor and the microstructural integrity of white matter as observed through neuroimaging [89]. Further studies are needed to investigate the possible connections of the found proteins with the brain microstructure and functioning.

Large-scale proteomic studies with plasma samples can present multiple challenges that need to be addressed to generate robust and meaningful results. For instance, protein expression in plasma is dynamic, and both interindividual and sample variability can be notable. Furthermore, plasma proteomic studies differ in the pipelines and methods used due to a lack of standard protocols [19]. Additional challenges include ensuring consistent sample handling and processing [90], normalizing data, correcting signal drift and batch effects [35, 91], accounting for biological variability [92], improving reproducibility [93], and managing the resource-intensive nature of such studies [94]. Despite these limitations, proteomics remains a powerful tool that can contribute to better diagnostics of mental health [95, 96]. The major constraint in this study is that the proteomic data was only obtained once for each participant. This one-time snapshot of a dynamically evolving organism makes it challenging to conclusively link the identified biomarkers to the investigated p-factor. The true nature of these associations is also hard to determine based solely on these data. These correlations could be the outcome of underlying biological processes or inherent biological traits of the participants, which might simultaneously influence both protein abundance and the p-factor (the observed behavior). Alternatively, the changes in protein abundance and the p-factor could be causally related, either as a cause or as an effect. Mental conditions may cause divergent effects on the abundance of plasma proteins, as demonstrated in the study by Shin and colleagues [69]. Further investigations will benefit from the inclusion of the disorder symptoms into the p-factor, which is missing from the score used in the present manuscript. These limitations suggest that a more detailed investigation into the various components of the p-factor may be needed to identify more specific biomarkers.

The strength of this study lies in its large cohort size, and the use of modern proteomics methods, which made it possible to obtain proteome profiles of hundreds of individuals, each comprising hundreds of plasma protein abundancies. This large scale allows us to identify common patterns in the proteomes of individuals with high and low p-factor values. While the changes in plasma abundancies of some of the proteins were previously reported, other proteins were linked to a vulnerability to the development of general psychopathology for the first time. Our research utilized the FT12 cohort, a large and thoroughly characterized population-based cohort with a broad range of measured characteristics, making the proteomic data gathered in this study an invaluable resource for future exploration and analysis.

## Conclusions

The study suggests that examining plasma proteomic profiles makes it possible to elucidate the biological processes related to the p-factor, which may inform the future development of novel screening, diagnostic, or therapeutic strategies for mental disorders. The results revealed proteins with common cellular functions connected to the p-factor, reflecting the general psychopathology. However, further studies are needed to examine the identified proteins and their potential as biomarkers for mental health dysfunction. In the future, utilization of the p-factor may also have implications for the development of interventions targeting common underlying factors that contribute to multiple forms of mental disorders. By addressing these shared factors, interventions could potentially be more effective in improving mental health outcomes across a range of disorders.

### Supplementary information

Supplementary figure 1

Supplementary figure 2

Supplementary file 1

Supplementary figure captions

Supplementary tables

## Data Availability

The data analyzed in this study is subject to the following licenses/restrictions: The FT12 data is not publicly available due to the restrictions of informed consent. Requests to access these datasets should be directed to the Institute for Molecular Medicine Finland (FIMM) Data Access Committee (DAC) (fimmdac@helsinki.fi) for authorized researchers who have IRB/ethics approval and an institutionally approved study plan. To ensure the protection of privacy and compliance with national data protection legislation, a data use/transfer agreement is needed, the content and specific clauses of which will depend on the nature of the requested data.
