# Supplementary figures and images for: Proteomic insights into mental health status: plasma markers in young adults
Source: Transl Psychiatry. 2024 Jan 24;14:55. doi: 10.1038/s41398-024-02751-z (PMC10808121; doi:10.1038/s41398-024-02751-z)

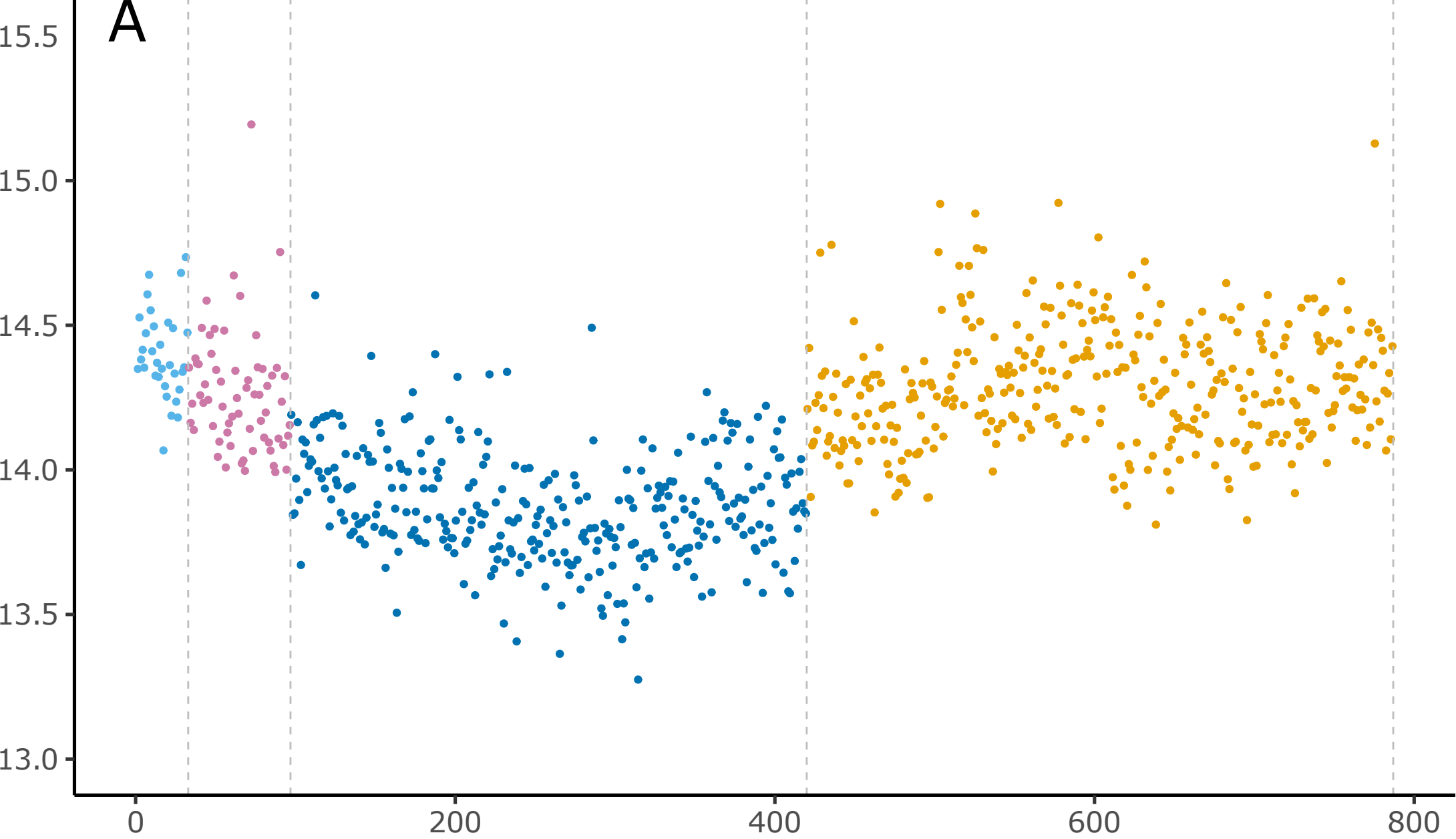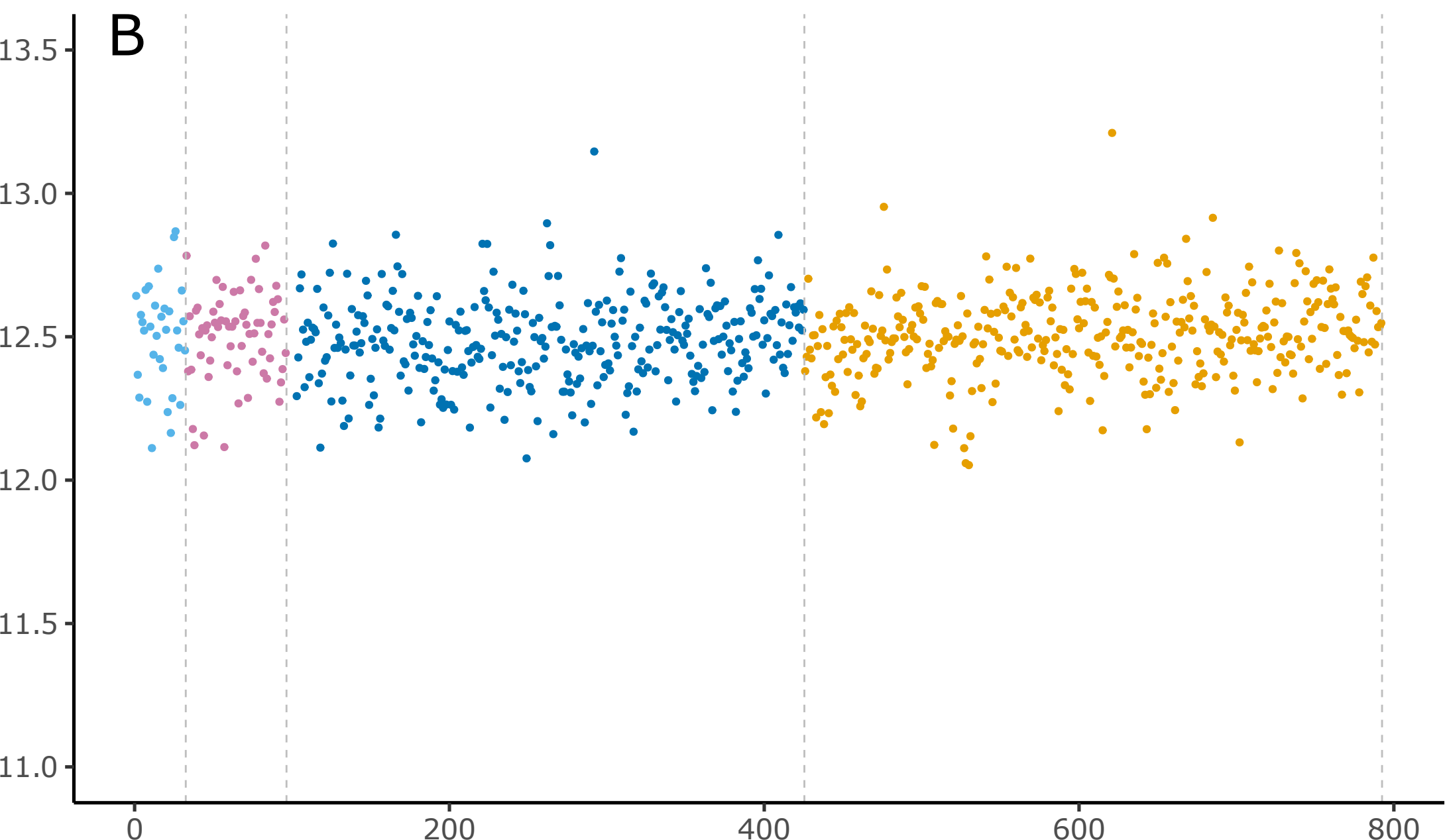

Supplement: Supplementary file 1 — Supplementary figure 1 [file 41398_2024_2751_MOESM1_ESM.pdf]

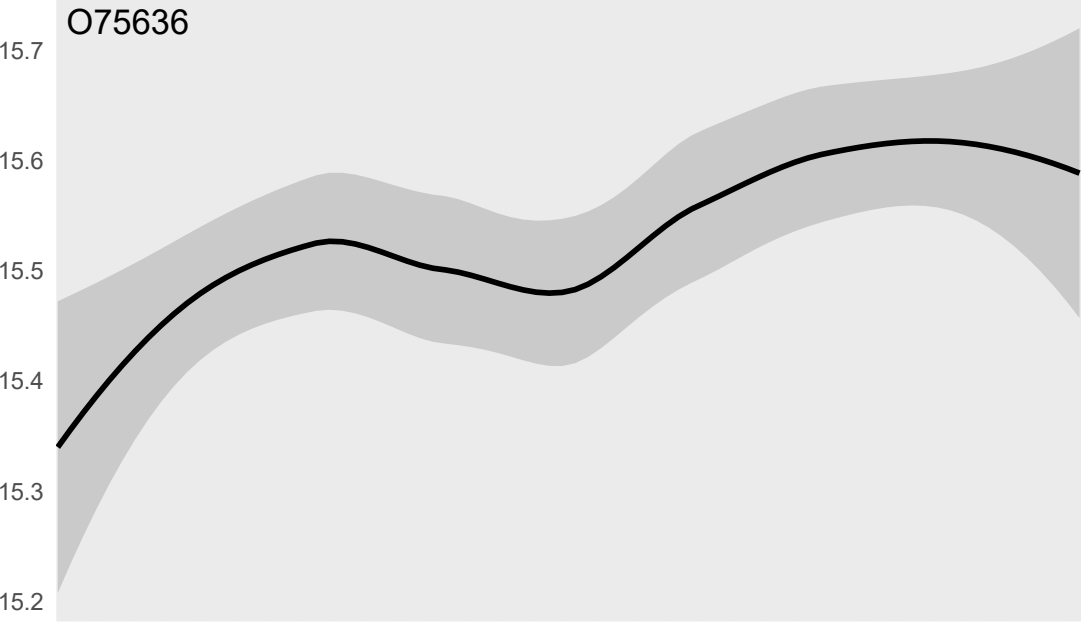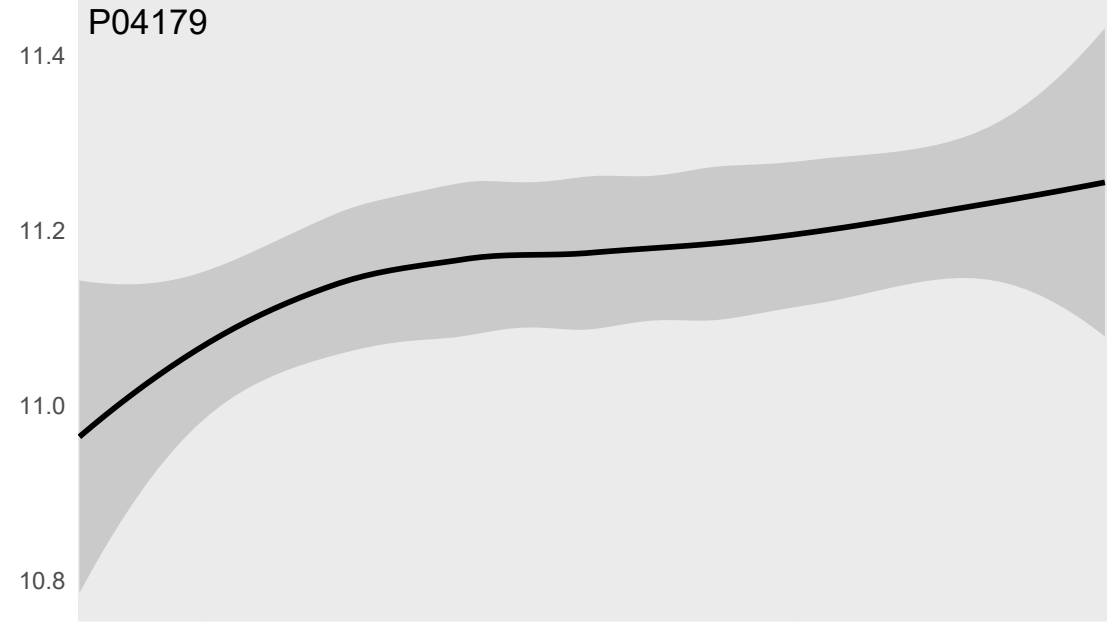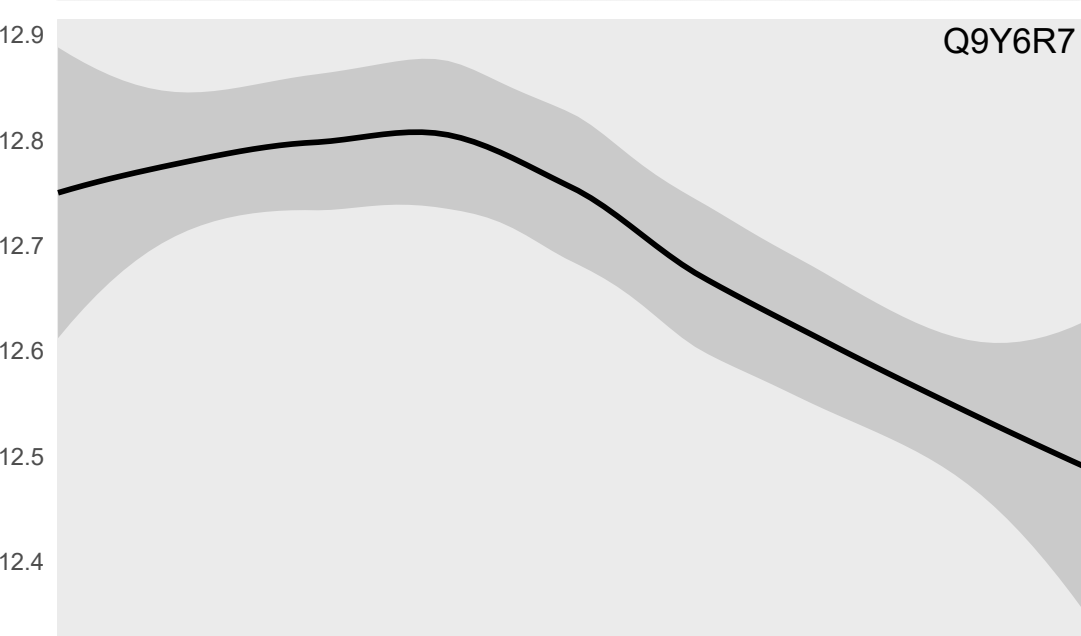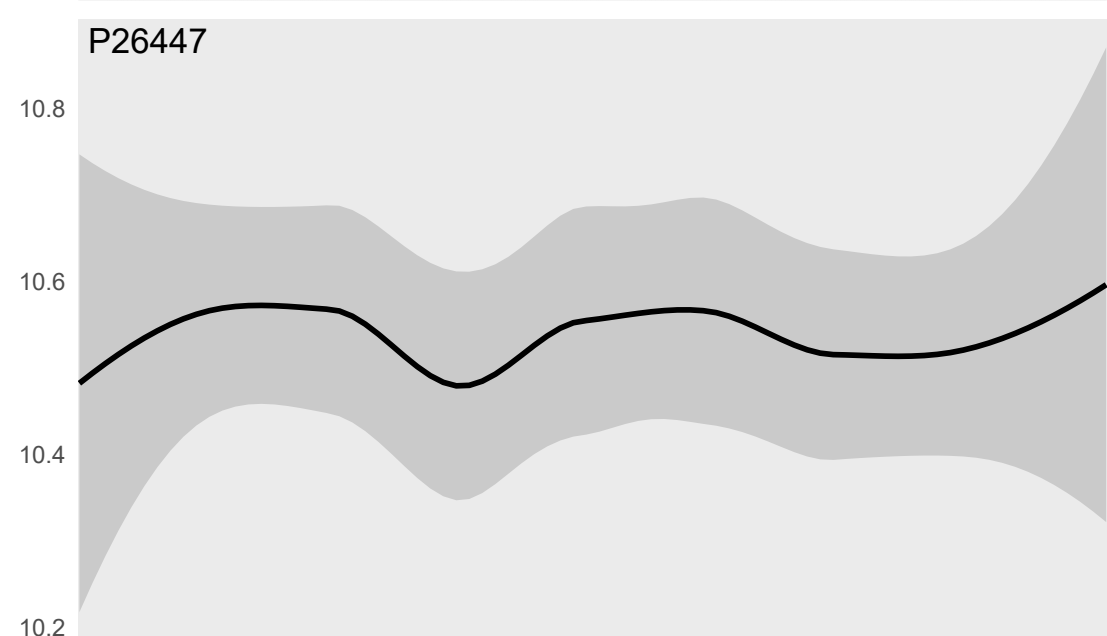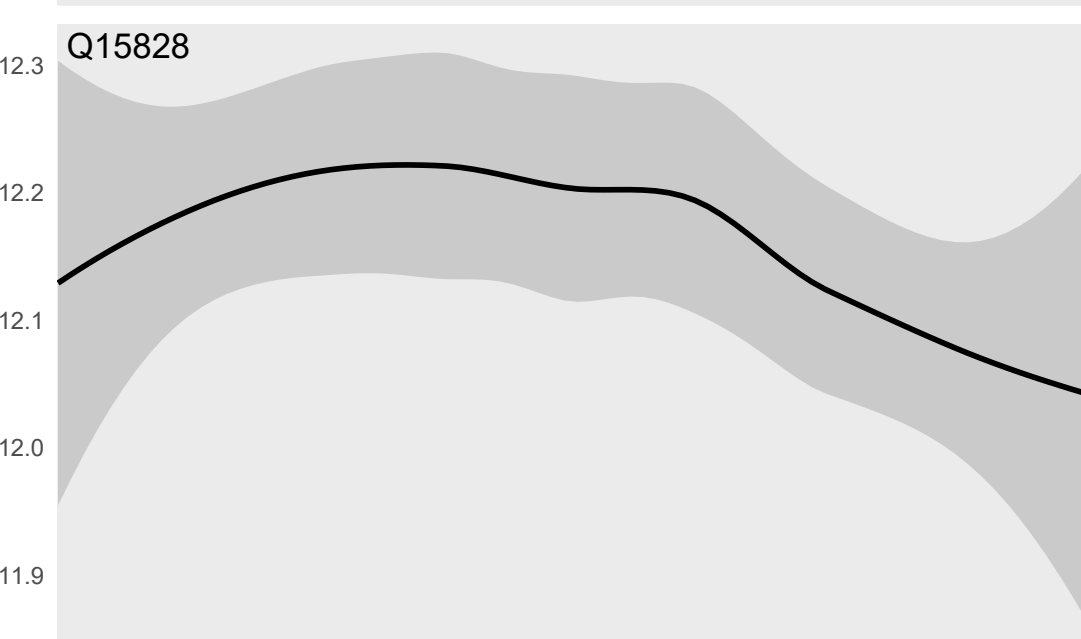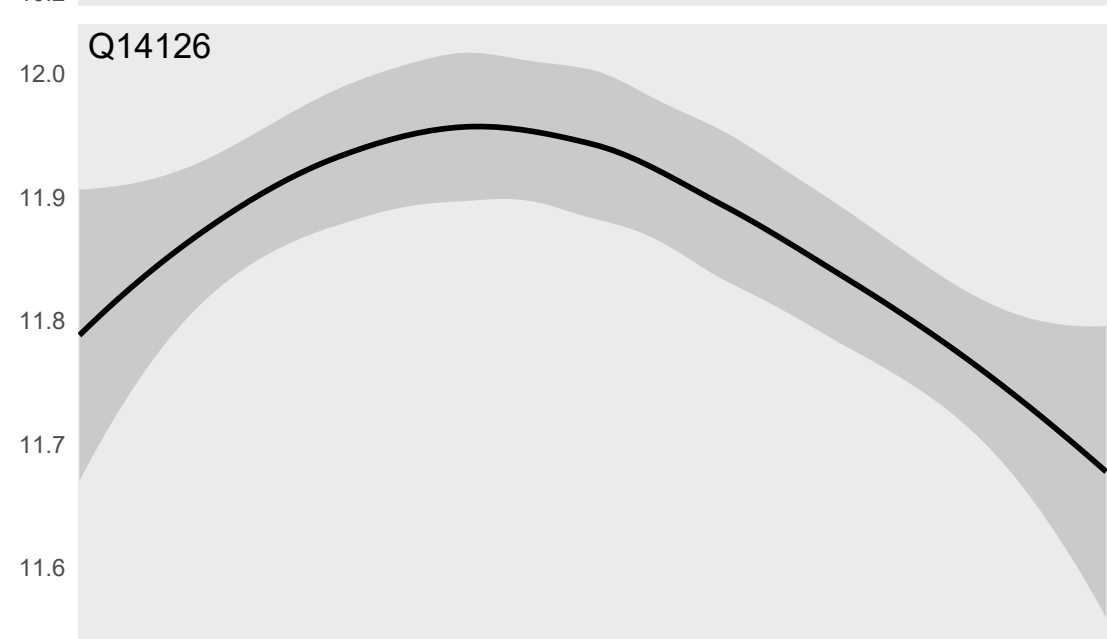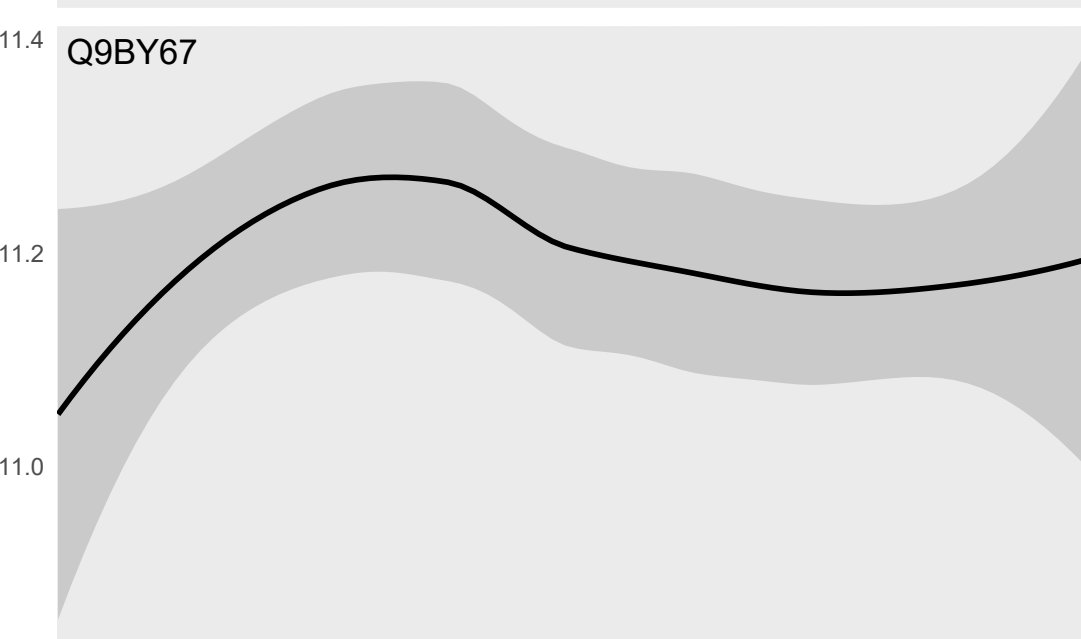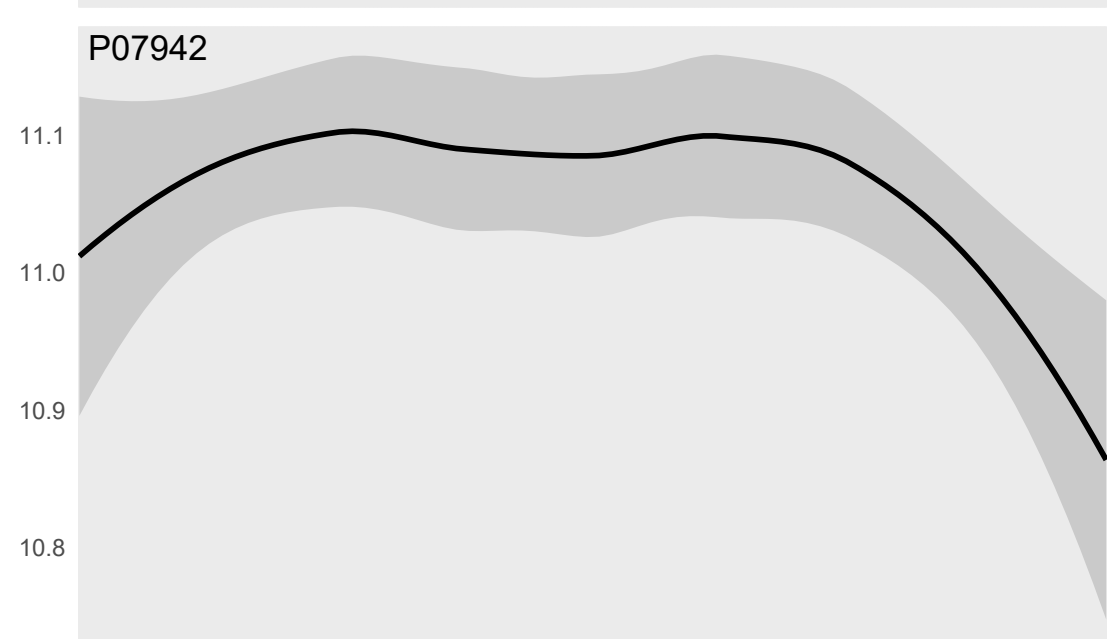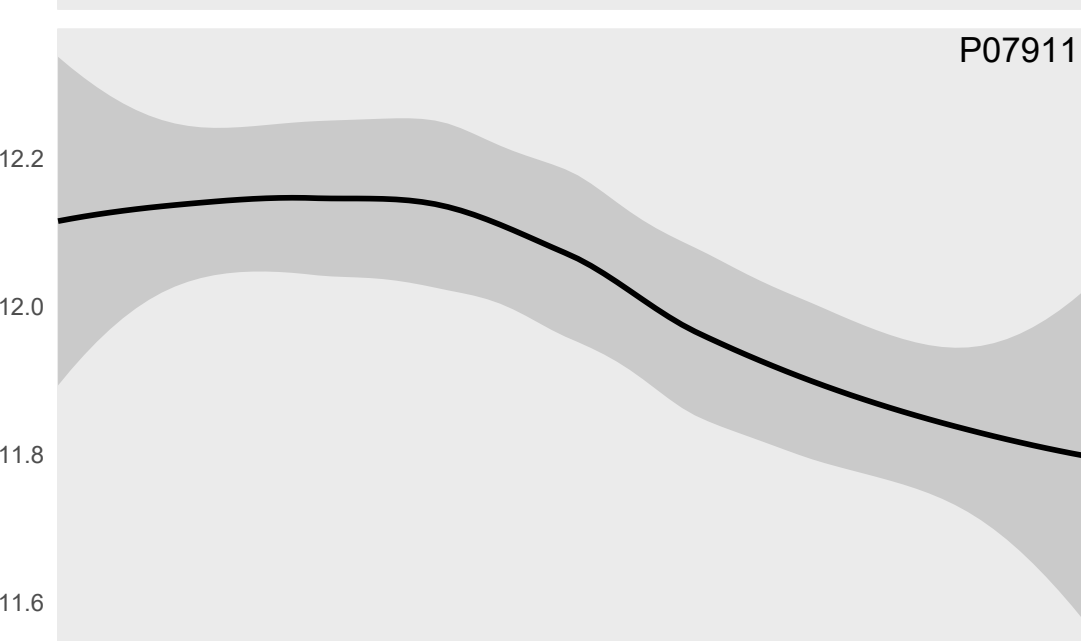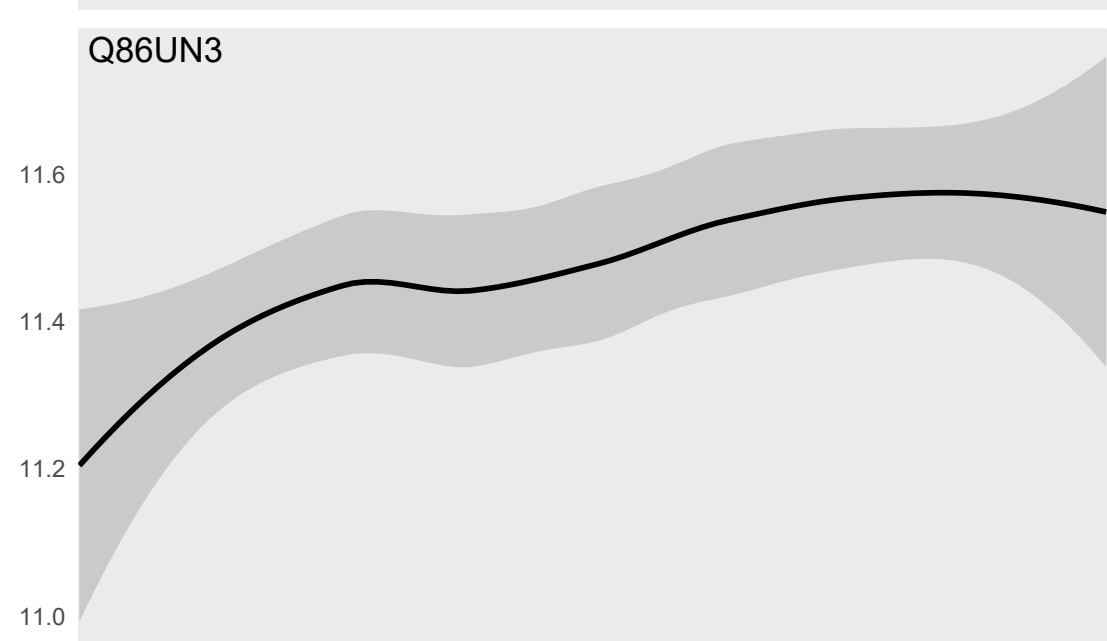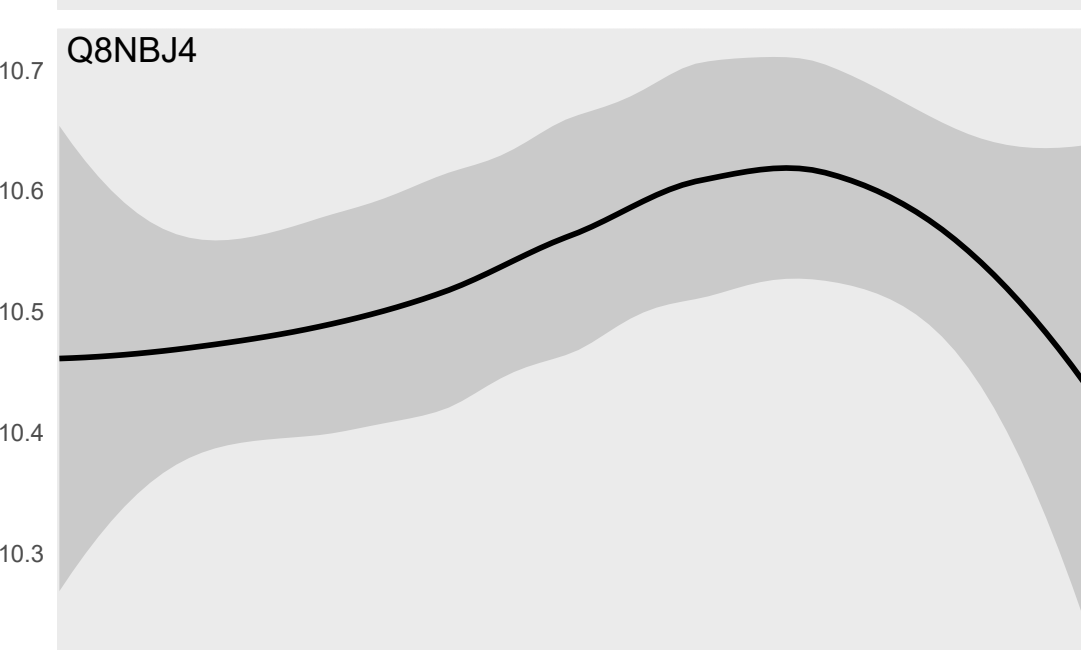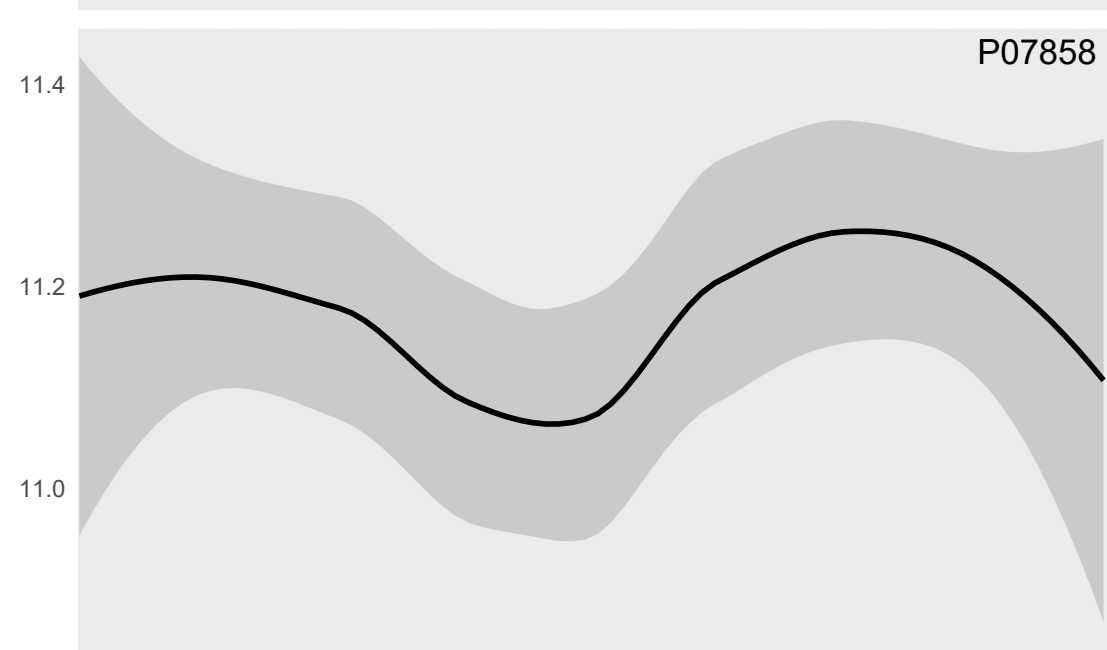

Supplement: Supplementary file 2 — Supplementary figure 2 [file 41398_2024_2751_MOESM2_ESM.pdf]
