# Supplementary material for: Proteomic insights into mental health status: plasma markers in young adults
Source: Transl Psychiatry. 2024 Jan 24;14:55. doi: 10.1038/s41398-024-02751-z (PMC10808121; doi:10.1038/s41398-024-02751-z)
Supplement: Supplementary file 4 — Supplementary figure captions [file 41398_2024_2751_MOESM4_ESM.docx]

Supplementary figure 1. Plots of mean intensity values for each sample. A) the original values B) after drift and batch correction. Each dot represents the mean intensity values for single samples and the samples are plotted chronologically. The batches are coloured and separated with dotted grey lines. The first two batches coincide with separate launches of the instrument. The latter batches are from the same launch, in-between the batches the instrument was cleaned, which led to a noticeable increase in the mean intensity.

Supplementary figure 2. Plots of abundance for proteins with significant non-linear associations with the p-factor. The lines were constructed using geom_smooth function of the ggplot2 package. The abundance scales are different for each plot. X-axis represent participants sorted by the p-factor, lowest p-factor being on the left
