# Supplementary material for: Proteomic insights into mental health status: plasma markers in young adults
Source: Transl Psychiatry. 2024 Jan 24;14:55. doi: 10.1038/s41398-024-02751-z (PMC10808121; doi:10.1038/s41398-024-02751-z)
Supplement: Supplementary file 5 — Supplementary tables [file 41398_2024_2751_MOESM5_ESM.docx]

Supplementary table 1. The results of additional linear and nonlinear analyses using subsets with different cutoffs on the number of missing values

|  | ***20% missing values*** | | ***40% missing values*** | | ***60% missing values*** | | ***80% missing values*** | | ***98% missing values*** | |
| --- | --- | --- | --- | --- | --- | --- | --- | --- | --- | --- |
| Protein ID | ***Linear q-value*** | ***Nonlinear q-value*** | ***Linear q-value*** | ***Nonlinear q-value*** | ***Linear q-value*** | ***Nonlinear q-value*** | ***Linear q-value*** | ***Nonlinear q-value*** | ***Linear q-value*** | ***Nonlinear q-value*** |
| O75636 | 0.200 | 0.022 | 0.009 | 0.024 | 0.265 | 0.026 | 0.279 | 0.030 | 0.288 | 0.030 |
| P98160 | 0.005 | 0.055 | < 0.001 | 0.061 | 0.009 | 0.069 | 0.011 | 0.071 | 0.014 | 0.069 |
| Q9Y6R7 | 0.004 | 0.006 | < 0.001 | 0.005 | 0.007 | 0.005 | 0.009 | 0.006 | 0.011 | 0.007 |
| P23142 | 0.005 | 0.061 | < 0.001 | 0.073 | 0.008 | 0.084 | 0.010 | 0.083 | 0.012 | 0.088 |
| P04179 | 0.706 | 0.001 | 0.506 | 0.001 | 0.851 | < 0.001 | 0.806 | < 0.001 | 0.794 | < 0.001 |
| P26447 | 0.052 | 0.040 | - | - | - | - | - | - | - | - |
| Q15828 | 0.020 | 0.040 | < 0.001 | 0.035 | 0.027 | 0.040 | 0.034 | 0.048 | 0.034 | 0.042 |
| Q14126 | 0.061 | 0.007 | 0.001 | 0.010 | 0.129 | 0.009 | 0.138 | 0.011 | 0.169 | 0.013 |
| Q9BY67 | 0.315 | 0.001 | 0.273 | 0.001 | 0.890 | < 0.001 | 0.946 | < 0.001 | 0.931 | < 0.001 |
| P07942 | 0.200 | 0.029 | 0.009 | 0.031 | 0.265 | 0.036 | 0.279 | 0.042 | 0.288 | 0.037 |
| P07911 | 0.004 | 0.007 | < 0.001 | 0.012 | 0.008 | 0.015 | 0.010 | 0.023 | 0.012 | 0.022 |
| Q86UN3 | 0.695 | 0.007 | 0.296 | 0.012 | 0.708 | 0.004 | 0.706 | 0.008 | 0.685 | 0.013 |
| Q8NBJ4 | 0.917 | 0.019 | 0.690 | 0.025 | 0.891 | 0.040 | - | - | 0.805 | 0.015 |
| P07858 | 0.513 | 0.007 | 0.192 | 0.010 | 0.549 | 0.001 | 0.564 | 0.003 | 0.592 | 0.007 |
| A5PLL7 | - | - | 0.367 | < 0.001 | 0.774 | < 0.001 | 0.732 | 9.245 | 0.730 | < 0.001 |
| A6NLJ0;Q8NCU7 | - | - | 0.647 | 0.020 | 0.847 | 0.011 | 0.899 | 0.006 | 0.902 | 0.003 |
| P12110 | - | - | - | - | 0.911 | 0.006 | 0.934 | 0.029 | 0.939 | 0.029 |
| P24043 | - | - | - | - | 0.834 | 0.005 | 0.843 | 0.006 | 0.832 | 0.008 |
| O43895 | - | - | - | - | - | - | 0.190 | 0.011 | 0.216 | 0.019 |
| Q5HYA8 | - | - | - | - | - | - | 0.322 | 0.048 | 0.341 | 0.036 |
| P07988 | - | - | - | - | - | - | 0.039 | 0.173 | 0.034 | 0.132 |
| Q13576 | - | - | - | - | - | - | - | - | 0.947 | 0.019 |
| P13497 | - | - | - | - | - | - | - | - | 0.889 | 0.036 |
| Q5PSV4 | - | - | - | - | - | - | - | - | 0.716 | 0.008 |
| Q96HY6 | - | - | - | - | - | - | - | - | 0.242 | 0.034 |

Only significant q-values are presented in the table.

Supplementary table 2. The results of the enrichment analysis using STRINGdb

| ***Category*** | ***Term*** | ***Protein number*** | ***Genes in background*** | ***Protein IDs*** | ***p-value*** | ***FDR*** | ***Description*** |
| --- | --- | --- | --- | --- | --- | --- | --- |
| COMPARTMENTS | GOCC:0005576 | 11 | 2035 | LAMB1,FCN3,CST6,FBLN1,RTN4RL2,CTSB,S100A4,HSPG2, GOLM1,UMOD,SOD2 | < 0.001 | < 0.001 | Extracellular region |
| COMPARTMENTS | GOCC:0005615 | 8 | 985 | LAMB,FCN3,FBLN1,CTSB,S100A4,HSPG2,UMOD,SOD2 | < 0.001 | < 0.001 | Extracellular space |
| COMPARTMENTS | GOCC:0070062 | 5 | 368 | CTSB,S100A4,HSPG2,UMOD,SOD2 | < 0.001 | 0.003 | Extracellular exosome |
| COMPARTMENTS | GOCC:0031012 | 4 | 253 | LAMB1,FBLN1,S100A4,HSPG2 | < 0.001 | 0.010 | Extracellular matrix |
| Component | GO:0005615 | 13 | 3195 | LAMB1,DSG2,FCN3,CST6,FBLN1,RTN4RL2,CTSB,S100A4,HSPG2,GOLM1,UMOD,SOD2,FCGBP | < 0.001 | < 0.001 | Extracellular space |
| Component | GO:0070062 | 11 | 2099 | LAMB1,DSG2,CST6,FBLN1,RTN4RL2,CTSB,S100A4,HSPG2, UMOD,SOD2,FCGBP | < 0.001 | < 0.001 | Extracellular exosome |
| Component | GO:0031012 | 7 | 527 | LAMB1,FCN3,FBLN1,RTN4RL2,CTSB,S100A4,HSPG2 | < 0.001 | < 0.001 | Extracellular matrix |
| Component | GO:0062023 | 6 | 396 | LAMB1,FCN3,FBLN1,CTSB,S100A4,HSPG2 | < 0.001 | < 0.001 | Collagen-containing extracellular matrix |
| Component | GO:0005604 | 3 | 96 | LAMB1,FBLN1,HSPG2 | < 0.001 | 0.008 | Basement membrane |
| Function | GO:0005201 | 4 | 119 | LAMB1,FBLN1,HSPG2,UMOD | < 0.001 | 0.005 | Extracellular matrix structural constituent |
| TISSUES | BTO:0001491 | 12 | 5020 | LAMB1,DSG2,FCN3,CADM1,FBLN1,CTSB,S100A4,HSPG2, GOLM1,UMOD,SOD2,FCGBP | < 0.001 | 0.008 | Viscus |
| TISSUES | BTO:0004850 | 4 | 170 | LAMB1,DSG2,HSPG2,FCGBP | < 0.001 | 0.008 | Bone marrow cell |
| Keyword | KW-0732 | 11 | 3233 | LAMB1,DSG2,FCN3,CST6,CADM1,FBLN1,RTN4RL2,CTSB,HSPG2,UMOD,FCGBP | < 0.001 | < 0.001 | Signal |
| Keyword | KW-0325 | 11 | 4349 | LAMB1,DSG2,FCN3,CST6,CADM1,FBLN1,RTN4RL2,CTSB,HSPG2,GOLM1,UMOD | < 0.001 | 0.004 | Glycoprotein |
| Keyword | KW-0964 | 7 | 1818 | LAMB1,FCN3,CST6,FBLN1,CTSB,HSPG2,UMOD | < 0.001 | 0.022 | Secreted |
| Keyword | KW-1015 | 9 | 3304 | LAMB1,FCN3,CST6,CADM1,FBLN1,RTN4RL2,CTSB,HSPG2,UMOD | < 0.001 | 0.022 | Disulfide bond |
| Keyword | KW-0106 | 5 | 875 | DSG2,FCN3,FBLN1,S100A4,HSPG2 | < 0.001 | 0.031 | Calcium |
| Keyword | KW-0424 | 2 | 30 | LAMB1,HSPG2 | < 0.001 | 0.031 | Laminin EGF-like domain |
| Keyword | KW-0084 | 2 | 39 | LAMB1,HSPG2 | < 0.001 | 0.037 | Basement membrane |
| Keyword | KW-0245 | 3 | 229 | FBLN1,HSPG2,UMOD | 0.001 | 0.046 | EGF-like domain |
| InterPro | IPR000742 | 5 | 233 | LAMB1,FBLN1,HSPG2,UMOD,FCGBP | < 0.001 | < 0.001 | EGF-like domain |
